# Perceptual correlates of successful body–prosthesis interaction in lower limb amputees: psychometric characterisation and development of the Prosthesis Embodiment Scale

**DOI:** 10.1038/s41598-020-70828-y

**Published:** 2020-08-26

**Authors:** Robin Bekrater-Bodmann

**Affiliations:** Department of Cognitive and Clinical Neuroscience, Central Institute of Mental Health, Medical Faculty Mannheim, Heidelberg University, Square J5, 68159 Mannheim, Germany

**Keywords:** Psychology, Health care, Medical research, Signs and symptoms

## Abstract

Prostheses are used to at least partly restore the body after limb amputation. Making the user accepting the prosthetic device as part of his or her body, i.e., inducing prosthesis embodiment, has been identified as major aim of prosthetic treatment. However, up to now, there is no consensus about the psychometric nature of prosthesis embodiment in limb amputees. In the present study, 118 unilateral lower limb amputees using a prosthesis were asked to complete an online questionnaire targeting prosthesis embodiment. Principal axis factoring revealed the factor structure of prosthesis embodiment, i.e., *Ownership/Integrity*, *Agency*, and *Anatomical Plausibility*, which resembles the embodiment structure previously identified for normally-limbed participants. The majority of amputees achieved prosthesis embodiment as assessed with the final version of the newly developed *Prosthesis Embodiment Scale*. Internal consistency was excellent, and test–retest reliability was satisfying, while the instrument was also sensitive for new prosthetic equipment. Validation on the basis of relationships to prosthesis satisfaction and adjustment to prosthesis use was performed. The *Prosthesis Embodiment Scale* could be a valuable tool for the assessment of perceptual correlates of successful body–prosthesis interaction in rehabilitative and research contexts, the latter which might further benefit from the comparability of psychometrically evaluated data.

## Introduction

The amputation of a limb represents the most serious breach of one’s physical integrity. For centuries, prosthetic devices have been used to—at least partly—restore the body after limb amputation. Since the beginnings of prosthetics, prosthetic treatment predominantly addressed aspects of functionality and cosmetics^1^. Psychological outcomes of using a prosthesis, however, only recently attracted increased scientific attention.

For example, low prosthesis satisfaction, including factors such as appearance and functionality, has been identified as crucial predictor for prosthesis rejection in upper limb amputees^2^, and has further been related to body image disturbances in lower limb amputees^3^. Remarkably, there is evidence for perceptual interaction between the amputee’s body and the prosthesis. Thus, prosthesis users tend to overestimate their residual limb’s length^4^, and other results suggest that prostheses expand the users’ peripersonal space encompassing the residual limb^5^. These results indicate that the prosthesis can be represented at least as *extension* of the body; however, the question remained open whether the artificial limb can also be perceived as integral *part* of the body, that is, whether or not true embodiment for the prosthesis can be achieved. This difference is anything but trivial, since it has been proposed that prosthesis embodiment, rather than perceiving the device as mere tool attached to the body, is an important perceptual correlate of successful body–prosthesis interaction^1,6,7^, and might thus constitute a crucial marker of prosthesis satisfaction and adjustment to prosthesis use (e.g.,^1,8,9^).

The basis for the scientific investigation of processes underlying embodiment dated back more than 20 years ago. In their seminal study, Botvinick and Cohen^10^ showed that a visible rubber hand can be perceived as belonging to a normally-limbed participant’s body by synchronous visuotactile stimulation together with the participant’s hidden hand, known as the so-called rubber hand illusion (RHI). Subsequent studies, using the RHI paradigm or its derivatives, revealed the universality of the underlying processes, since the induction of embodiment is not restricted to the upper limbs, but can also be elicited for other parts of the body, including the lower limbs, by using multimodal sensory or sensorimotor stimulation applied to a corresponding artificial counterpart (e.g.,^10–16^; for a review see^17^). Embodiment has been related to behavioural (e.g.,^10,18^), peripheral physiological (e.g.,^19,20^), and central neurophysiological measures (e.g.,^12^), which validated subjective experiences. It has been assumed that the processes underlying embodiment are fundamental also for everyday self-experiences and the formation of a bodily sense of self^21^. Accordingly, embodiment has been proposed to be a basic neurobiological mechanism involved in the discrimination between oneself and others^21^ and might have a self-protective function^22^. The pivotal nature of the phenomenon is emphasized by evidence for embodiment-like behaviour even in non-primate mammals^23^.

What, however, is the psychometric structure of embodiment in humans? Longo et al.^18^ experimentally induced the RHI in a sample of more than one hundred normally-limbed participants and asked them to complete a questionnaire targeting their experiences. By applying principal component analysis, the authors particularly identified an embodiment component which was consisting of three separable sub-dimensions: *ownership* refers to the experience that the artificial limb is belonging to one’s own body; *location* refers to perceived spatial congruence between the felt and the observed limb or the felt and observed touch; and *agency* refers to the sense of being in control of the artificial limb. The embodiment component was selectively related to behavioural measures of the illusion^18^, suggesting validity of psychometric findings. The dimensions of embodiment have been repeatedly identified as crucial contributors to bodily self-experiences (e.g.,^24–26^); however, whether or not the psychometrics of embodiment assessed in short-term and experimental contexts apply for long-term and real-life prosthesis use, remains unknown^27^.

Impaired physical integrity, by all means, does not suspend the normal processes underlying embodiment. As Ehrsson et al.^28^ reported, even upper limb amputees can be induced to perceive the RHI when the residual limb is tactilely stimulated in synchrony with an observed artificial limb. Accordingly, it has been proposed that the RHI could be an experimental model for prosthesis embodiment^7,29^. Empirical evidence for perceptual prosthesis embodiment in amputees, however, remains scarce. Murray^6^, for instance, conducted qualitative interviews on phenomenological aspects of prosthesis use in 35 participants with upper or lower limb loss (eight of which reported congenital limb absence). The author identified several themes related to embodied prosthesis experiences, which in the amputee sample involved topics such as adjustment to prosthesis use, altered body mechanics, attention spent to the device, and the prosthesis being a tool versus a part of the body. Wijk and Carlsson^30^, on the other hand, found evidence for the perception of prosthesis agency, but not prosthesis ownership, in a content analysis of 13 interviews with prosthesis-using participants, six of which with congenital limb absence. The qualitative nature of these studies in combination with small and heterogenous samples, however, does not allow for eventual conclusions about the phenomenon. In their sample of more than 200 upper and lower limb amputees using a prosthesis, Giummarra et al.^31^ identified 29% who embodied their device. However, this categorization was mainly based on qualitative reports on perceived superposition of the phantom limb on the prosthesis, and perceptual correlates of embodiment were not systematically assessed. Since there is evidence that phantom limb awareness is neither a necessary nor sufficient condition for prosthesis embodiment^1,6^, the study might thus crucially underestimate the prevalence of the phenomenon. Moreover, due to unavailability of a validated instrument targeting prosthesis embodiment, previous studies used inconsistent measures including aspects not related to bodily self-experiences in the narrow sense (e.g.,^1,32^), crucially impeding the comparability and interpretability of findings.

The present study aimed at evaluating the transferability of psychometrics reported for embodiment experiences in normally-limbed participants to limb amputees using prostheses. Thus, the study had two main aims: (a) describing the psychometric structure of prosthesis embodiment, and (b) developing an instrument for the reliable and valid assessment of prosthesis embodiment experiences from a patient-oriented point of view. For this purpose, a sample of 118 lower limb amputees was asked to complete a literature-based 13-item questionnaire targeting perceptual correlates of body–prosthesis interaction. Item selection mainly based on previously reported items^18^ that appeared to be particularly suitable for inferring the underlying latent dimensions of embodiment in normally-limbed participants. The resulting *Prosthesis Embodiment Scale* was further evaluated regarding its reliability in terms of internal consistency and test–retest stability, and validity in terms of prosthesis satisfaction and adjustment to prosthesis use.

## Results

### Initial item analysis

Analysis of the 13 items (see Table 6) is provided in Table 1. Items showed a wide range of difficulty which is desirable for a psychometric instrument. However, items #4 and #10 showed discriminatory power below the acceptable value of 0.50^33^ each, creating doubts whether they sufficiently discriminate between different levels of prosthesis embodiment. Item #4 (*Resemblance*) refers to visual characteristics of the prosthesis, requiring the comparison of the device’s appearance with an actual limb. Based on the item analysis, the appropriateness of this item appears doubtful for leg amputees, which could be due to the fact that many lower limb prostheses, especially the modular ones, have rather a technical visual appearance not really resembling an actual leg. Accordingly, the item might target visual evaluation of the prosthesis cover rather than ownership experiences in the narrow sense, and thus, only unlikely reflects a reliable measure of prosthesis embodiment. Similarly, item #10 (*Touch*) might apply only for a minority of amputees who potentially show vision-touch synesthesia^34^, i.e., the rare condition of perceiving a tactile sensation via a visually applied touch. Since both items probably induce invalid variance in the data and their relationship to embodiment remains unknown, they were excluded from further analyses.

**Table 1:** Descriptive item analysis.

Item #	Item label	Mean^a^	SD	Median^a^	IQR	Diff.	DP
1	Ownership	1.37	1.88	2.00	3.00	0.73	0.83
2	Belongingness	1.90	1.55	2.00	1.00	0.82	0.73
3	Affiliation	1.39	1.79	2.00	2.00	0.73	0.83
4	Resemblance	− 0.97	1.96	− 1.50	4.00	0.34	0.45
5	Self-observation	− 0.25	2.01	0.00	4.00	0.46	0.58
6	Integrity	1.03	1.93	2.00	3.00	0.67	0.79
7	Completeness	1.15	1.81	2.00	3.00	0.69	0.82
8	Location	2.19	1.15	3.00	1.00	0.87	0.52
9	Posture	1.15	1.59	2.00	2.00	0.69	0.62
10	Touch	− 0.17	2.09	0.00	4.00	0.47	0.46
11	Volition	2.14	1.22	2.50	1.00	0.86	0.55
12	Controllability	2.33	1.06	3.00	1.00	0.89	0.55
13	Vividness	1.40	1.64	2.00	2.00	0.73	0.75

*SD* standard deviation, *IQR* interquartile range, *Diff.* item difficulty, *DP* discriminatory power of item.

^a^Item scores potentially range from − 3 to + 3.

### Principal axis factoring

Principal axis factoring (PAF) was conducted on the eleven remaining items with oblique rotation. An initial analysis was run with Kaiser’s criterion (factor eigen values > 1). All extracted communalities were ≥ 0.30 (*M* = 0.65; *SD* = 0.21). The Kaiser–Meyer–Olkin (KMO) measure verified sampling adequacy for the analysis, KMO = 0.87, and all KMO values for individual items were ≥ 0.76, which is well above the acceptable limit of 0.50^35^. Bartlett’s test of sphericity, *χ*^2^_55_ = 1,011.85, *p* < .001, indicated that correlations between items were sufficiently large for conducting PAF. Two factors had eigen values over Kaiser’s criterion and in combination explained 65.37% of the variance. However, visual inspection of the scree plot indicated that there could be a third factor which missed Kaiser’s criterion. The results of subsequent parallel analysis confirmed presence of a third factor (eigen value of 0.47, simulated *M* = 0.33, 95% confidence interval (*CI*) = 0.23–0.44) which together with the other two factors explained 70.25% of the variance.

Therefore, another PAF was performed, by specifically targeting a three-factor solution. The level of extracted communalities improved (all ≥ 0.38; *M* = 0.70; *SD* = 0.16; mean values ≥ 0.70 have been assumed to be acceptable^36^). The factor loadings after rotation (pattern matrix) indicated that ten of the eleven items load sufficiently high on a single factor each (all factor loadings ≥ 0.62), while concurrently loading low on the other two factors (all factor loadings between − 0.15 and 0.37). The items (#1, #2, #3, #5, #6, and #7) that clustered on factor 1 target experiences of both ownership and physical integrity, so that this factor was labelled *Ownership/Integrity*. The items (#11 and #12) that clustered on factor 2 represented *Agency*. Items (#8 and #9) loading on factor 3 referred to anatomical plausibility of body–prosthesis structure in space, so that this factor was termed *Anatomical Plausibility* hereafter. Item #13 (*Vividness*) did not load sufficiently high on any factor (all factor loadings between 0.30 and 0.37).

**Figure 1:**
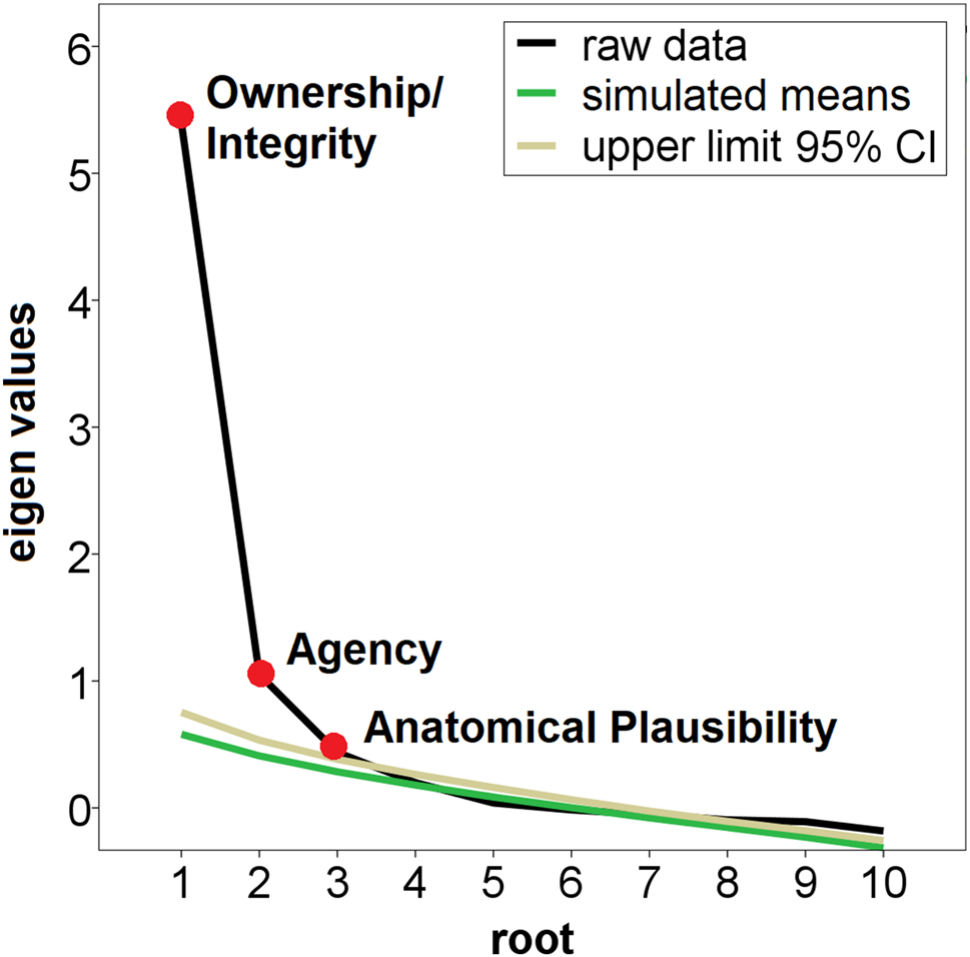
Scree plot of the final principal axis factor analysis (3-factor solution), with simulated means and their 95% confidence interval (CI, upper limit) resulting from parallel analysis. Labels of embodiment dimensions are provided.

Running a final PAF by excluding item #13 (all extracted communalities ≥ 0.38 (*M* = 0.71, *SD* = 0.17); KMO measure for sampling adequacy = 0.85; all KMO values for individual items ≥ 0.72; Bartlett’s test of sphericity, χ^2^_45_ = 896.00, *p* < .001) revealed an identical three-factor solution (71.10% explained variance) which was also confirmed by parallel analysis (eigen value of 0.44 for the third factor, simulated *M* = 0.28, 95% *CI* = 0.19–0.38; the scree plot with simulated eigen values from the parallel analysis is given in Fig. 1). The factor loadings (pattern matrix) of this final PAF are given in Table 2, where also the data of the structure matrix can be found.

**Table 2:** Pattern matrix for the final principal axis factor analysis with oblique rotation (3-factor solution).

Item #	Item label	Factors		
		Ownership/integrity	Agency	Anatomical plausibility
1	Ownership	**0.855** (0.903)	0.042 (0.488)	0.044 (0.572)
2	Belongingness	**0.854** (0.804)	0.088 (0.470)	− 0.156 (0.386)
3	Affiliation	**0.902** (0.916)	0.012 (0.471)	0.013 (0.560)
5	Self-observation	**0.629** (0.610)	− 0.077 (0.251)	0.034 (0.388)
6	Integrity	**0.796** (0.835)	0.021 (0.438)	0.047 (0.533)
7	Completeness	**0.665** (0.840)	− 0.006 (0.424)	0.297 (0.695)
8	Location	0.025 (0.478)	− 0.044 (0.219)	**0.789** (0.790)
9	Posture	0.087 (0.534)	0.219 (0.440)	**0.558** (0.680)
11	Volition	− 0.043 (0.495)	**0.996** (0.993)	0.059 (0.348)
12	Controllability	0.069 (0.495)	**0.872** (0.899)	− 0.024 (0.294)
Explained variance (%)		54.32	11.34	5.44

Given are the factor loadings as regression coefficients (correlation coefficients of the structure matrix are further provided in parentheses). Bold fond indicates the items’ factor affiliation based on regression coefficients.

Further analyses examining the factor structure of the final set of items provided support for the 3-factor structure underlying prosthesis embodiment. These results can be found in the supplement (Supplementary results: Factor structure; Confirmatory factor analyses; Figures S1 and S2).

### Prosthesis Embodiment Scale scoring

**Figure 2:**
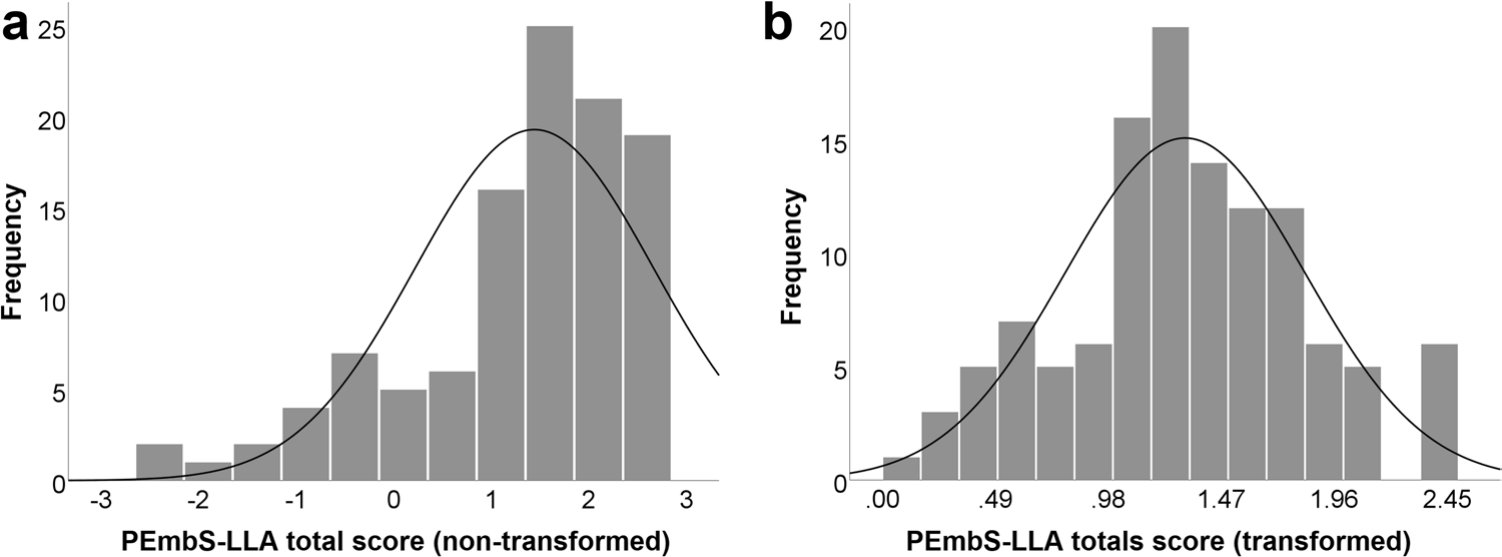
Distribution of the total score of Prosthesis Embodiment Scale for Lower Limb amputees (PEmbS-LLA) for raw (**a**) and transformed data (**b**).

The ten items identified in the principle factor analysis constituted the *Prosthesis Embodiment Scale for Lower Limb Amputees* (PEmbS-LLA). The PEmbS-LLA total score was defined as the mean of these items. A majority of participants (85.75%) affirmed perceptual embodiment for their prosthesis, as indicated by positive PEmbS-LLA total scores. The PEmbS-LLA total score was characterized by substantial negative skewness and kurtosis, and thus, its distribution significantly differed from normality according to the Shapiro–Wilk test (*W*_118_ = 0.91, *p* < .001). Reverse square root transformation was performed which normalized distribution (*W*_118_ = 0.98, *p* = .17). Note that the transformed PEmbS-LLA total score (re-reversed) range is 0 to √6 (≈ 2.45). Raw and transformed PEmbS-LLA total scores are given in Table 3, and its distributions are further visualized in Fig. 2. The PEmbS-LLA sub-scale scores can be found in the supplement (Supplementary results: Scoring of the the PEmbS-LLA sub-scales; Table S1).

**Table 3:** Characteristics of raw and transformed total scores for the Prosthesis Embodiment Scale for Lower Limb Amputees.

	Prosthesis Embodiment Scale total score	
	Raw data^a^	Transformed data^b^
Mean	1.44	1.31
*SD*	1.22	0.52
Median	1.60	1.27
*IQR*	1.33	0.59
Skewness	− 1.13	0.08
*SE* skewness	0.22	0.22
Kurtosis	1.00	− 0.03
*SE* Kurtosis	0.44	0.44

*SD* standard deviation, *IQR* interquartile range, *SE* standard error.

^a^Non-transformed data, potential range from − 3 to + 3.

^b^Reversed square root transformed data, back reversed, potential range from 0 to √6 (≈ 2.45).

### Reliability: internal consistency and test–retest stability

For the PEmbS-LLA, Cronbach’s alpha was 0.91, suggesting excellent internal consistency^37^. Internal consistency for the PEmbS-LLA sub-scales can be found in the supplement (Supplementary results: Reliability: internal consistency and test–retest stability of the Prosthesis Embodiment Scale’s sub-scales).

Test–retest reliability of the PEmbS-LLA was evaluated by a second assessment at least 2 months after first data collection. This was done in two independent samples: *temporal stability* of prosthesis embodiment was estimated by two assessments (T1 and T2) with the identical prosthesis (*n* = 32); and *contextual stability* was examined by two assessments with different devices each, due to new prosthetic equipment in the meantime (*n* = 16). For this purpose, the difference in prosthesis embodiment (transformed PEmbS-LLA total score) between the first and second assessment (T2 minus T1) was compared against 0. This difference as well as the absolute individual amount of variation were further compared between both samples. Furthermore, Pearson correlations were performed using the transformed data of T1 and T2.

Prosthesis embodiment neither significantly changed by time (mean (*M*) = − 0.04, standard deviation (*SD*) = 0.29, *t*_31_ = − 0.85, *p* = .40) nor by context (*M* = − 0.13, *SD* = 0.44, *t*_15_ = − 1.17, *p* = .26), and both samples did not differ significantly from each other (*t*_21.80_ = 0.70, *p* = .49). The absolute variation in prosthesis embodiment between both assessments, however, differed significantly in the two samples (*t*_46_ = − 2.34, *p* = .024), with prosthesis embodiment being more stable over time (*M* = 0.21, *SD* = 0.21) than across contexts (*M* = 0.36, *SD* = 0.26). This suggests that new prosthetic equipment induces more changes in prosthesis embodiment than the mere passing of time with the same device, but there is no general tendency in which direction (less or more) prosthesis embodiment will shift.

Temporal stability was good for the PEmbS-LLA (*r*_30_ = 0.84). Partial correlation controlling for time between first and second assessment did not change temporal stability (*r*_29_ = 0.84, *p* < .001). Contextual stability was lower (*r*_14_ = 0.55, *p* = .03). Again, controlling for time between both assessments did not change the contextual stability (*r*_13_ = 0.55, *p* = .03). The comparison between Fisher’s *Z*-transformed correlation coefficients for both stability measures revealed significantly stronger temporal compared to contextual stability (*Z* = 1.81; one-tailed *p* = .04).

Test–retest stability correlations showed a comparable pattern for the *Ownership/Integrity* sub-scale, but were only partly comparable for the *Agency* and *Anatomical Plausibility* sub-scales (see Supplementary results: Reliability: internal consistency and test–retest stability of the Prosthesis Embodiment Scale’s sub-scales).

### Further validation

Since the distributions of the sub-scales of prosthesis satisfaction and adjustment to prosthesis use significantly differed from normality (*W*_118_ = 0.76–0.97, all *p* ≤ .011), Spearman correlations with the transformed PEmbS-LLA total score data were performed. All measures showed significant medium-sized (*ρ* between 0.34 and 0.47, all *p*_Bonf_ < .001) correlations in the expected direction. Statistical details are provided in Table 4. The pattern of results was comparable for the PEmbS-LLA sub-scales (see Supplementary results: Further validation; Table S2; particularly note the additional and specific correlation between *Agency* and *advanced locomotor capability* as well as the specific correlation between *Ownership/Integrity* and *social adjustment*).

**Table 4:** Correlation of the Prosthesis Embodiment Scale’s total score and selected variables related to prosthesis acceptance.

	*Mdn* (*IQR*)	Correlation with PEmbS-LLA total score^a^	*p*_Bonf_
Aesthetic prosthesis satisfaction	2.00 (0.67)	0.342	< 0.001
Functional prosthesis satisfaction	2.40 (0.60)	0.389	< 0.001
General adjustment	3.80 (0.60)	0.424	< 0.001
Social adjustment	3.60 (1.00)	0.386	< 0.001
Adjustment to limitation	2.80 (1.20)	0.467	< 0.001

*Mdn* median, *IQR* interquartile range, PEmbS-LLA Prosthesis Embodiment Scale for Lower Limb Amputees, *p*_Bonf_ Bonferroni-corrected *p* value.

^a^Spearman correlation coefficient (*ρ*).

## Discussion

The present study sought to elucidate the perceptual correlates of successful body–prosthesis interaction in terms of prosthesis embodiment in lower limb amputees. More specifically, the study aimed at (a) the description of the psychometric structure of prosthesis embodiment and (b) the development of an instrument for the reliable and valid assessment of prosthesis embodiment experiences. Based on previous findings^18^, the *Prosthesis Embodiment Scale for Lower Limb Amputees* (PEmbS-LLA; final German and English versions are provided in the supplement (Supplementary materials); note the diverging item numbering) was developed and its sub-dimensions, i.e., *Ownership/Integrity*, *Agency*, *Anatomical Plausibility*, were identified using a factor-analytical approach. Data suggest that most amputees experience some level of prosthesis embodiment, as indicated by positive PEmbS-LLA scores. The PEmbS-LLA showed good to excellent reliability in terms of internal consistency and test–retest stability with the same prosthetic device. However, the PEmbS-LLA also seems to be sensitive to new prosthetic equipment. Content and construct validity appear to be given, based on the factor-analytical approach and meaningful relationships to prosthesis satisfaction and adjustment to prosthesis use. The present study’s results might be of utmost theoretical and practical interest, since they empirically suggest for the first time that lower limb prosthesis embodiment (a) can be perceptually achieved in most amputees, (b) is qualitatively comparable to experimentally induced embodiment experiences in normally-limbed participants, (c) represents a temporally stable, but contextually transient phenomenon, and (d) is related to positive rehabilitative outcomes.

Previous studies on prosthesis embodiment are scarce^7^, and the few published reports either collected qualitative data in small and heterogenous samples (e.g.,^6,30^), categorized participants based on questionable criteria (e.g.,^31^), or used inconsistent measures to assess the phenomenon (e.g.,^32,38^). Therefore, it remained unclear whether embodiment of a prosthesis could be perceived at all^7,27^. The present study’s results are indicative of a high prevalence of prosthesis embodiment in lower limb amputees: about 86% of participants affirmed the presence of prosthesis embodiment, as indicated by positive PEmbS-LLA total scores. This suggests that—although there is considerable variance in interindividual experiences—the majority of lower limb amputees can at least perceptually achieve some level of prosthesis embodiment. Previous research emphasized the dimensional nature of body perception^39^, suggesting that specific aspects of prosthesis embodiment may distribute on a continuum and not being discrete as to be present or absent^40^. Thus, it remains an open question at which perceptual level—as assessed with the PEmbS-LLA—substantial prosthesis embodiment has been reached.

Exploratory factor analysis revealed a 3-factor structure underlying prosthesis embodiment. Additional statistical approaches in terms of hierarchical structure analysis^41^ and confirmatory factor analysis (see supplement) supported the rejection of a single-factor prosthesis embodiment model in favour of a multi-factorial model. Although both a 2-factor and a 3-factor model were statistically supported by confirmatory factor analysis, results from hierarchical structure analysis and parallel analysis suggest validity of a 3-factor model of prosthesis embodiment. This interpretation of results not least fits theoretical considerations insofar that the 3-factor structure of the PEmbS-LLA reproduces that structure previously identified for the RHI^18^, which is remarkable given the differences in participants’ physical integrity (amputated vs normally-limbed), the targeted limb (leg vs arm), the context (real-life vs experimental), and temporal characteristics (long-term vs short-term). The conformity of factorial findings suggests perceptual universality and high robustness of the underlying processes, and further supports previous authors who proposed substantial similarity in the processes underlying both experimentally-induced and prosthesis embodiment^29,42,43^. However, the findings regarding the factor structure underlying prosthesis embodiment have to be replicated and further validated in prospective studies before conclusiveness can be assumed.

There is an ongoing debate whether experiences related to prosthesis use reflect tool-mediated extension of the body representation or true perceptual embodiment of the device. Although tool use changes central body and peripersonal space representations (e.g.,^44,45^; for a review see^46^), perceptual embodiment cannot be achieved for tools^27,47,48^. This might be related to different neurophysiological mechanisms underlying the representation of the body and body extensions. For experienced upper limb prostheses users, it has recently been shown that pictures of prosthetic hands activate hand-selective visual brain areas^49^. Upper limb prosthesis use has further been associated with structural alterations in posterior parietal cortex^50^, a region which has also been related to embodiment experiences in normally-limbed participants^12^ and upper limb amputees^51^. Whether or not these neural underpinnings are also involved in prosthesis embodiment, however, remains open and has to be elucidated in prospective studies.

Remarkably, prosthesis embodiment appears to be a temporally stable perception. This is in line with results regarding the temporal stability of experimental embodiment experiences^52^. Interestingly, contextual stability of prosthesis embodiment appeared to be significantly weaker, suggesting a crucial mediating role of the prosthetic device for the induction of embodiment experiences. Results related to contextual stability (*r* = 0.55), that is, invariance of prosthesis embodiment across different prosthesis contexts, indicates that about 30% of observed variance in prosthesis embodiment (determination coefficient of 0.30) could be explained by stable individual responses to the device in terms of a perceptual trait. The difference to temporal stability (*r* = 0.84; determination coefficient of 0.71) further suggests that prosthetic features could account for another 40% of the variance. What could these prosthesis features be? Bekrater-Bodmann et al.^52^ showed that contextual variations reducing multimodal sensory conflicts can enhance the intensity of embodiment, and the combination of sensory and motor feedback has been identified as having additive effects on behavioural proxies of embodiment in experimental contexts^16^. Evidence for amputees, however, is scarce. In (multiple) case or small-sample studies on upper^53,54^ and lower limb amputees^55^ it has been shown that appropriate multimodal sensory or sensorimotor feedback is capable to enhance prosthesis embodiment, and neurotactile feedback, alone or in combination with congruent visual stimulation, appeared to be particularly promising^56,57^. However, it has to be noted that the sample sizes for analysing temporal and contextual stability in the present study were small and its validity has to be further explored in prospective studies using proper sample sizes (cf.,^58^), before of which the present test–retest stability findings have to be considered exploratory and thus preliminary.

Once the requirements of prosthesis embodiment have been identified, the knowledge can also be used to improve rehabilitative outcomes of prosthesis use. Imaizumi et al.^38^ showed that embodied arm prostheses are positively associated with stabilized body posture, and Gouzien et al.^32^ found a significant positive relationship between peripersonal space extent in a reachability judgement task and upper limb prosthesis embodiment. However, the investigated amputee samples were small in both studies (*n* = 9 and 12, respectively). Including a large sample of amputees, the results of the present study revealed that lower limb prosthesis embodiment is positively associated with prosthesis acceptance in terms of prosthesis satisfaction and adjustment to prosthesis use, suggesting that prosthesis embodiment might contribute to everyday psychosocial functioning and could reduce the risk of prosthesis rejection^2^. However, the causal direction of the associations remains unknown, and has to be further investigated in future experimental studies. Furthermore, behavioural validation of rehabilitative outcomes, such as functional capacity or gait analyses, is required, so that the present results can only be considered preliminary validation. If confirmed, the PEmbS-LLA might be of rehabilitative value, since it could reveal perceptual deficits associated with prostheses use, at an early stage of prosthetic equipment or in the long run, which otherwise might be easily overlooked.

Several limitations of the present study have to be considered. Firstly, it has to be noted that the term *prosthesis embodiment* has previously been used in wider concepts, including cognitive, cultural, social, or gender perspectives^1,27^; the present study, however, uses the term exclusively to refer to the phenomenology of incorporating the prosthesis into the amputee’s body representation^27^, and thus sought to investigate only one of several embodiment dimensions. With the introduction of the PEmbS-LLA, the present study provides a psychometrically evaluated and theoretically sound reliable and valid instrument which can be used in prospective studies on prosthesis embodiment in order to enhance the conclusiveness of results; though, future studies have to investigate the relationship of different embodiment dimensions to each other in order to specify their influence on prosthesis acceptance. Secondly, factoring highly depends on the item pool entered into the analyses^18^, and this pool was limited due to the present study purposes. The limited number of items resulted in two PEmbS-LLA sub-scales composed of only two items each (*Agency* and *Anatomical Plausibility*), which has been proposed to be potentially problematic^35^. These sub-dimensions further contributed only marginally to the total amount of explained variance. Moreover, good to excellent reliability was particularly evident for the PEmbS-LLA total score. Based on these findings, the author of the present study recommends using the PEmbS-LLA total score for prospective research or clinical purposes, unless there are weighty scientific or practical-clinical reasons for using its sub-scale scores (for instance, for specific research questions or when participants are not able to walk with their prosthesis). Thirdly, the present study’s validation of prosthesis embodiment solely relies on subjective data targeting prosthesis acceptance; however, the gold standard requires a multitrait-multimethod approach^59^. Previous studies associated experimentally induced embodiment in the RHI paradigm with altered proprioception (e.g.,^10,18^) or peripheral physiological anxiety responses to applied threats (e.g.,^19^). However, these measures have been developed for normally-limbed participants and often require the existence of a physical counterpart^7^, so that future studies need to develop new objective measures to evaluate the level of prosthesis embodiment in limb amputees. Recently, Di Pino et al.^9^ reported a case of an upper limb amputee equipped with a prosthesis with intraneural sensory feedback. Behavioural experiments suggested an extension of peripersonal space over a training period of 1 month, representing an implicit measure of prosthesis embodiment. Applying such tasks to lower limb amputees, and combining them with measures of perceptual prosthesis embodiment as assessed with the PEmbS-LLA, might be viable to substantiate the findings of the present study. Fourthly, before general validity of the present results can be assumed, they should be replicated in an independent sample of lower limb amputees. This should also be done for more representative samples; for instance, the reason for amputation in the present sample showed a bias to accidents (almost 70%) which is strikingly different to the general population of lower limb amputees^60^. Moreover, it remains open whether the present results apply also for other prosthesis-using populations such as persons with upper limb amputation or with congenital limb deficiency. Since the PEmbS-LLA based on results of RHI embodiment in normally-limbed participants^18^ and showed high consistency to these findings, it can be assumed that the experiences are universal, which probably allows for population-specific adaptations of the *Prosthesis Embodiment Scale*. Prospective studies, however, have to confirm the general applicability of the new instrument, which is also true for its translations to other languages than German.

In conclusion, the present study’s results suggest that prosthesis embodiment is a highly prevalent condition achieved in lower limb amputees. Qualitatively, prosthesis embodiment resembles experiences reported for experimental investigations in normally-limbed participants. The underlying perceptual mechanisms appear to represent a universal and temporally stable trait; however, characteristics of the prosthesis might crucially contribute to embodiment experiences. The newly developed *Prosthesis Embodiment Scale* might help to validly and reliably evaluate the perceptual correlates of successful body–prosthesis interaction in clinical and scientific contexts; the rehabilitative value of prosthesis embodiment has yet to be determined in the future.

## Methods

### Procedure

Lower limb amputees were recruited using the PHANTOMMIND data base (initial description by Bekrater-Bodmann et al.^61^; permission to use the data base was obtained from the responsible authorities), flyers distributed to professionals working in prosthetic rehabilitation centres, and calls via print and social media. Inclusion criteria were (a) an age between 18 and 80 years, (b) unilateral acquired major lower limb amputation, (c) owning a prosthesis to compensate for limb loss, (d) access to the internet, and (e) sufficient comprehension of the German language. Congenital limb deficiency was an exclusion criterion in the present study. Recruited amputees were screened via telephone interview for eligibility to participate. Afterwards, the consent form was sent postally or via email. The study was approved by the ethics review board of the Medical Faculty Mannheim, Heidelberg University, and adhered to the Declaration of Helsinki in its current form.

Written informed consent was obtained from all participants. After return of the signed consent form, a pseudonymized link to an online questionnaire battery (in German language) was provided. The battery was implemented in Gorilla Experiment Builder (https://gorilla.sc/) and was composed of questionnaires on demographics, amputation and prosthesis information, phantom phenomena, as well as instruments on prosthesis acceptance, psychosocial functioning, and body image. Unless items were optional, participants were forced to respond. For the present study’s purposes, prosthesis embodiment data as well as information on demographics, amputation, prosthesis use and satisfaction, and adjustment to the prosthesis were used. For evaluating the stability of prosthesis embodiment experiences (see below), the questionnaire targeting prosthesis embodiment was postally sent once again to a sub-sample of participants along with a reply-paid envelope on average ~ 3 months after participation in the online study.

### Sample description

From May to December 2019, 126 participants were recruited from which eight had to be excluded due to missing data in prosthesis embodiment items. Thus, 118 prosthesis-using unilateral major leg amputees (75.42% male; mean (*M*) age = 56.30 years, standard deviation (*SD*) = 9.83, range 30–76 years) were enrolled in the present study. On average, the amputation dated back *M* = 28.93 years (*SD* = 15.00, range 4 months to 64 years). Further clinical details of the sample are provided in Table 5.

**Table 5:** Amputation-related characteristics of the present sample.

Characteristics	n	%
Left-sided amputation	69	58.47
Amputation of dominant limb^a^	43	39.81
**Level of amputation**		
Foot amputation	4	3.39
Transtibial amputation	58	49.15
Knee exarticulation	3	2.54
Transfemoral amputation	53	44.92
Hip exarticulation or hemipelvectomy	0	0.00
**Reason for amputation (multiple responses allowed)**		
Accidents	82	69.49
Injuries	17	14.41
Cancer	17	14.41
Infections	14	11.86
Peripheral vascular diseases	8	6.78
Other reasons	10	8.47
**Phantom limb sensations**		
Presence of phantom limb awareness^b^	71	60.17
Presence of phantom limb pain^b^	60	50.85

*n* number.

^a^108 valid cases (ten missing data due to not-remembering) for the question *Which leg did you use to kick an object, for example a ball, prior to amputation?*

^b^In the last four weeks

Corrected for longer breaks (> 1 month), the participants used a prosthesis for *M* = 28.75 years (*SD* = 14.94, range 0–64 years; one missing data). Participants were further asked for certain features of their current main prosthesis, defined as the prosthesis that is used most of the time, if more than one prosthesis is owned. The main prosthesis, compared to other potential prostheses, was used for *M* = 95.43% of the time (*SD* = 9.95, range 50–100%, four missing data). This prosthesis was used for *M* = 54.61 months (*SD* = 63.50, range 0–304 months). Based on the amputees’ information about the main prosthesis’ manufacturer and model, 44.92% of participants used a modular prosthesis, which represents the gold standard in prosthetic treatment for leg amputees^62^. However, a majority (54.69%) was not able to provide sufficient information about prosthesis manufacturer and/or model, suggesting a far higher percentage of modular prostheses. Two participants stated that they had an osseointegrated prosthesis. Almost all participants (92.37%, one missing data) used their main prosthesis on a daily basis, with 86.44% using it from morning to evening. Functional prosthesis use, defined as the averaged reported percentage of using the prosthesis during walking, standing, and sitting activities, was *M* = 91.89% (*SD* = 17.34, range 6–100%).

### Development of the Prosthesis Embodiment Scale: selection and adaptation of items

The development of the *Prosthesis Embodiment Scale for Lower Limb Amputees* (PEmbS-LLA) based on items identified by Longo et al.^18^ which have been empirically related to an underlying embodiment dimension in normally-limbed participants. For each of these items, an equivalent with as synonymous as possible content was generated, based on the present study author’s expertise in amputee and body perception research, discussions with professionals, and additional literature search. Items were pre-tested in an independent sample of eight subjects using a lower limb prosthesis and were found to be comprehensible. Original and adapted items are provided in Table 6. Note that each item was designed in a way that it could be applied to amputees with or without phantom limb awareness, i.e., with or without the sensation that the missing limb is still present. Participants were asked to complete the questionnaire with their donned main prosthesis, which they had to confirm beforehand. In contrast to the original scale^18^, the adapted scale was initially composed of three parts preceded by a short introduction each which asks the participant to observe or touch the prosthesis or walk through the room for a few seconds. Embodiment items were randomly presented in fixed groups, along with other items on prosthesis evaluation, which are not considered in the present study’s context. As in the original study^18^, each item was presented along with a seven-point Likert scale, ranging from − 3 (strongly disagree) to + 3 (strongly agree). Specific information about item selection and adaptation is provided below.

**Table 6:** Original and adapted items and its affiliation to hypothetical embodiment dimensions.

(Hypothetical) embodiment dimensions	Item #	Longo et al.^18^	Present study	Adapted items’ abbreviation
Ownership		During the block…	□ I have put on my prosthesis Please look at your prosthesis for about 60 s □ I have looked at my prosthesis for about 60 s and I am ready to continue	
	1	… it seemed like the rubber hand was my hand	The prosthesis is my leg	Ownership
	2	… it seemed like the rubber hand belonged to me	The prosthesis belongs to me	Belongingness
	3	… it seemed like the rubber hand was part of my body	The prosthesis is a part of my body	Affiliation
	4	… it seemed like the rubber hand began to resemble my real hand	The prosthesis resembles my intact leg in terms of skin tone, freckles or other visual features	Resemblance
	5	… it seemed like I was looking directly at my own hand, rather than at a rubber hand	I feel as if I was looking directly at my own leg, rather than at a prosthesis	Self-observation
	6	—	It feels as if I had two legs	Integrity
	7	—	My body feels complete	Completeness
Location	8	… it seemed like the rubber hand was in the location where my hand was	The prosthesis is in the location where I would expect my leg to be, if it was not amputated	Location
	9	… it seemed like my hand was in the location where the rubber hand was	The posture of the prosthesis corresponds to that of a real leg	Posture
			Please look at your prosthesis and touch it for about 10 s □ I touched my prosthesis for about 10 s and I am ready to continue	
	10	… it seemed like the touch I felt was caused by the paintbrush touching the rubber hand	I feel a touch sensation in the prosthesis	Touch
Agency			Please stand up and walk around the room for about 30 s □ I have walked around the room for about 30 s and I am ready to continue	
	11	… it seemed like I could have moved the rubber hand if I had wanted	The prosthesis is moving the way I want it to move	Volition
	12	… it seemed like I was in control of the rubber hand	I am in control of the prosthesis	Controllability
	13	—	The movement of the prosthesis feels like an actual movement	Vividness

*Ownership* Original items targeting ownership ask for experiences of belongingness of the artificial limb to the body as well as its visual resemblance with a real limb. The adaptation of items (#1–5) preserves this meaning. Item #4 (*Resemblance*) was further specified in order to avoid ambiguity^10^. Moreover, two additional items (#6 and #7, *Integrity* and *Completeness*) were introduced based on the following theoretical considerations: if ownership for the prosthesis is achieved, participants should feel as if they have two legs, and if so, they should feel as being physically complete. While these items do not make sense for normally-limbed participants in the RHI paradigm due to obvious reasons, in amputees, successful body–prosthesis interaction in terms of embodiment should be manifested in the perception of an intact body.

*Location* The PEmbS-LLA was developed in an attempt to be suitable for all limb amputees using a prosthesis, regardless of whether or not phantom limb awareness is reported. This is why the present instrument completely resigns items involving the interaction between the prosthetic device and the phantom. Original item #8 and #9, however, focus on perceived co-location of the real and the artificial limb, which in a word-for-word adaption for amputees would ask for co-location between the phantom and the prosthesis. Though, phantom limb awareness is characterized by substantial intra- and interindividual variance^63,64^ and it remains unclear whether or not this perceptual feature is interacting with prosthesis embodiment experiences^1,7,31^. In order to develop an instrument whose use is not limited to prosthesis users perceiving a phantom, the adapted items #8 (*Location*) and #9 (*Posture*) focus on the plausibility of anatomical aspects of the prosthesis in (peripersonal) space, which has been shown to play an important role in embodiment processes^65–67^. By all means, this does not exclude the possibility that these items also assess perceptual phantom-prosthesis interactions in those who experience a phantom limb. The adaptation of item #10 followed similar theoretical considerations. The original item asks for perceived touch referral from the actually stimulated real limb to the artificial one. In amputees, comparable tactile stimulation cannot be applied due to obvious reasons. However, the phenomenon of vision-touch synaesthesia, i.e., the tactile sensation of a merely visually applied touch, has been related to embodiment experiences in the RHI paradigm before^34^, and there is evidence that about 20–30% of amputees experience the related mirror-touch synaesthesia phenomenon (e.g.,^68,69^), that is, an observed touch to the amputees intact limb or another person’s limb is tactilely perceived by the amputee. Comparable effects could contribute to prosthesis embodiment as well. Thus, adapted item #10 (*Touch*) focus on the tactile sensation of touch visually applied to the prosthesis.

*Agency* While the original study^18^ assessed agency for the artificial limb in a static paradigm, for the amputee adaptation, participants were asked to make a few steps before responding to the agency-targeting items. This led to replacing the subjunctive in the original items by definitive statements about perceived controllability and volition in adapted items. Furthermore, an additional item (#13) asks for the vividness of movement^70^, which was not included in the original study, but has been shown to be of potential relevance in amputees^71^.

### Item analysis

Item analysis was applied to initially check for adequacy of used items. Means, standard deviations, medians, interquartile ranges as well as item difficulties and discriminatory power of items are provided. All analyses reported in the main text were performed with IBM SPSS v22.

### Principal axis factoring

Principal axis factoring (PAF) was performed to identify the factor structure underlying prosthesis embodiment. PAF was selected in favour of principal component analysis, since this method is better suited for psychological models due to considering residual and error variance in the data^72^. Furthermore, PAF does not require multivariate normal distribution^73^ which is often unlikely for psychological variables, and the effects of non-normal distribution on the number of extracted factors and the estimated loading matrix is negligible^74^. Although the PEmbS-LLA development based on previously reported psychometric findings^18^, for prostheses, the factor structure is yet unclear, so that initially exploratory factoring was applied; however, confirmatory factor analyses were performed post hoc (see below). Factor structure was evaluated based on extracted eigen values, visual inspection of the scree plot, and parallel analysis^75^ using raw data permutation (1,000 permutations, 95% confidence interval for simulated eigen values). PAF was conducted with oblique rotation (direct oblimin, delta = 0), allowing for an intercorrelated factor structure, which appears more plausible for psychological variables than orthogonal rotation resulting in independent factors. Maximum number of iterations was set to 20. Extracted communalities, Kaiser–Meyer–Olkin measure for sampling adequacy as well as for individual items, Bartlett’s test of sphericity, and explained variances are reported. For the final PAF, the factor loadings (pattern matrix and structure matrix) are provided. Conclusive factor loadings were defined as item loadings > 0.50 (regression coefficients as provided by the pattern matrix) on a single factor while showing lower loadings for the other factors^76^.

In order to give more in-depth insights into the nature of latent variables underlying the PEmbS-LLA, the hierarchical structure of the factors was further examined based on correlations among factor scores as path coefficients between adjoining factor hierarchies^41^, and confirmatory factor analyses were performed for evaluating the most meaningful factor solutions. Methodological details of these additional analyses are provided in the supplement (see Supplementary methods: Factor structure; Confirmatory factor analyses).

### Scoring of the Prosthesis Embodiment Scale and its sub-scales

The mean value of PEmbS-LLA items served as total score, and its distribution was evaluated using the Shapiro–Wilk test. If there was reason to assume significant deviation from normal distribution, transformation was considered.

### Reliability: internal consistency and test–retest stability

Internal consistency of the PEmbS-LLA was evaluated with Cronbach’s alpha. Internal consistency measures have been shown to be robust against non-normally distributed data in sample sizes n > 100^77^.

For test–retest stability testing purposes, 59 participants (50.00% of the sample, randomly selected) were re-contacted after initial participation and asked to complete the questionnaire targeting prosthesis embodiment a second time. Forty-one participants replied with valid data. Unexpectedly, nine subjects stated that they received new prosthetic equipment in the meantime (shaft bedding adjustment, module replacement, or new device). Since this new prosthetic context might have influenced prosthesis embodiment experiences, but also could provide important information about its stability, 107 participants were asked once again for potential new prosthetic equipment in the context of a bi-annual update about project progress several weeks later. Seven participants replied in the affirmative, and completed again the questionnaire targeting prosthesis embodiment. Thus, *n* = 32 amputees participated in the analysis of *temporal stability* (test–retest reliability with identical prostheses), while *n* = 16 participants were included in the analysis of *contextual stability* (test–retest reliability with different prostheses). Both samples were independent (no overlap). The mean time between first (T1) and second assessment (T2) for the temporal (*M* = 104.06 days, *SD* = 23.77, range 69–202 days) and contextual stability (*M* = 121.81 days, *SD* = 49.00, range 66–234 days) was more than 3 or 4 months, respectively, and at least 2 months for each participant. First, the difference between both assessments (T2 minus T1) was compared against 0 using one-sample *t* tests for each sample. This value was further compared between both samples using an independent *t* test. Additionally, the absolute amount of variation from T1 to T2 was compared between the groups, representing a measure of instability of prosthesis embodiment experiences. Furthermore, Pearson correlation coefficients^78^ between T1 and T2 were calculated. In order to control for differences in time between first and second assessment, partial correlations including the individual time between the two assessments were performed. Test–retest reliability coefficients between 0.80 and 0.95 are desired for the psychometric quality of scales^79^. Statistical comparison of stability correlation coefficients was performed by their transformation to Fisher’s *Z* scores^80^. If not stated otherwise, two-tailed *p* values are reported.

Statistical procedures for internal consistency and test–retest stability for PEmbS-LLA sub-scales can be found in the supplement (see Supplementary methods: Reliability and further validation of PEmbS-LLA sub-scales).

### Further validation

For further validation, clinically relevant measures related to prosthesis satisfaction and adjustment to prosthesis use were correlated with prosthesis embodiment as assessed by the PEmbS-LLA. The *Trinity Amputation and Prosthesis Experience Scales-Revised*^81^ (TAPES-R) is a widely used instrument developed to multidimensionally assess prosthesis acceptance in lower limb amputees. For the present study, a German translation of the TAPES-R was used (provided by the Center for Orthopedic and Trauma Surgery, Heidelberg University Hospital, Heidelberg, Germany). Scoring of selected TAPES-R scales based on the original authors’ guidelines. The *Aesthetic* (three items) and *Functional Satisfaction* (five items) sub-scales were used to assess prosthesis satisfaction [3-point response scale for each item: 1 (not satisfied); 2 (satisfied); 3 (very satisfied)]. For each sub-scale, the items were averaged, so that high scores are indicative of satisfaction with the respective feature (potential scores range from1 to 3 each). Furthermore, the sub-scales *General Adjustment*, *Social Adjustment*, and reversed *Adjustment to Limitation* (5 items each) were used. Each item was answered using a 4-point Likert scale ranging from 1 (strongly disagree) to 4 (strongly agree). Higher scores are indicative of successful adjustment to prosthesis use, potentially ranging from 1 to 4 for each sub-scale. Spearman correlations were applied (*p* values were Bonferroni-corrected for multiple testing, i.e., *p*_Bonf_).

Procedures for further validation of the PEmbS-LLA sub-scales can be found in the supplement (Supplementary methods: Reliability and further validation of PEmbS-LLA sub-scales).

## Supplementary information

Supplementary Information.

## Data Availability

The dataset analysed for the current study is available from the author on reasonable request.
