# Supplementary material for: Perceptual correlates of successful body–prosthesis interaction in lower limb amputees: psychometric characterisation and development of the Prosthesis Embodiment Scale
Source: Sci Rep. 2020 Aug 26;10:14203. doi: 10.1038/s41598-020-70828-y (PMC7450092; doi:10.1038/s41598-020-70828-y)
Supplement: Supplementary file 1 — Supplementary Information. [file 41598_2020_70828_MOESM1_ESM.pdf]

Perceptual correlates of successful body-prosthesis interaction in lower limb amputees: psychometric characterisation and development of the Prosthesis Embodiment Scale

Robin Bekrater-Bodmann

– Supplement –

Supplementary Methods

Factor structure

In order to further investigate the factor structure of the *Prosthesis Embodiment Scale for Lower Limb Amputees* (PEmbS-LLA), correlations among factor scores were used as path coefficients between adjoining factor hierarchies[1]. Factor scores were calculated using the regression method, which allowed for correlated scores, matching the oblique rotation approach reported for the exploratory factor analysis, and associations were assessed using Pearson correlations. Path coefficients of a hierarchically lower level resulting from a joint factor were further compared applying Fisher's Z transformation. If the relationship between a higher-order factor and its split lower-order subfactors differ, this would indicate that they share a different amount of variance, suggesting distinct latent variables. These analyses were performed using IBM SPSS v25.

Confirmatory factor analysis

Confirmatory factor analyses (CFA) were employed to compare different factor solutions (one, two, or three factors) of the PEmbS-LLA using the maximum likelihood approach (implemented in IBM AMOS, v26). In case of the 2-factor and 3-factor solutions, prosthesis embodiment was modelled as second-order factor. The fit for each model was increased by treating the covariance between two residuals of a single factor as free parameter (based on a modification index ≥ 10). The models' fit was evaluated by the χ^2 goodness-of-fit test, incremental fit indices, parsimony indices, and the root mean square error of approximation (RMSEA)[2]. The fit across models was compared using a χ^2 difference test[3,4].

Reliability and further validation of PEmbS-LLA sub-scales

Internal consistency of the PEmbS sub-scales was evaluated with Cronbach's alpha. In case of subscales composed of only two items, Spearman-Brown correlations were applied[5], whose correlation coefficient is called standardized Cronbach's alpha when used in this way. For stability testing purposes, Pearson correlations were performed for normalized

transformed scores (correlation coefficient *r*), and Spearman correlations for non-normalized transformed scores (correlation coefficient *ρ*).[6]

In addition to the *Trinity Amputation and Prosthesis Experience Scales – Revised*[7] (as described in the main text), the *Locomotor Capability Index*[8] (LCI) was implemented for validating the PEmbS-LLA sub-scales. The LCI is a widely used self-report instrument to validly assess perceived locomotor capacity (rather than actual performance) of prosthesis-using lower limb amputees. It measures basic and advanced locomotor capability with 7 items each. Amputees were asked to indicate whether they would be able to perform certain given everyday activities with the prosthesis. While the original LCI used a 4-point ordinal scale, in the present study the more recently proposed 5-point response scale[9] was used: 0 (no); 1 (yes, if someone helps me); 2 (yes, if someone is near me); 3 (yes, alone with ambulation aids); 4 (yes, alone without ambulation aids). In the present study, a German translation (performed by two bilingual professionals with psychological background) of the LCI was used, and the subscales' item responses were averaged (potential score range from 0 to 4 for both scales), with higher scores indicating higher locomotor capability. Correlations with PEmbS-LLA sub-scales were performed using Spearman correlations.

Supplementary Results

Factor structure

Figure S1 provides the hierarchical factor structure of the PEmbS-LLA. The relationship between the first unrotated factor and its second level's split factors was significantly different for 2/1 compared to 2/2 ($Z = 17.08$, $p < .001$). On the third hierarchy level, factor 2/1 split into the separate factors 3/1 (*Ownership/Integrity*) and 3/3 (*Anatomical Plausibility*), and again, both factors significantly differed in their relationship to factor 2/1 ($Z = 17.77$, $p < .001$), indicating that these factors represent distinct latent variables. The high relationships for *Ownership/Integrity* across the hierarchy further emphasized its important role for explaining variance in prosthesis embodiment, mirroring the results obtained in the exploratory factor analysis (see main text). *Agency* emerged as separate factor already at the second hierarchy

level. A 4-factor solution did not reveal any meaningful item-to-factor association, and was thus not considered.

Confirmatory factor analysis

Confirmatory factor analysis was employed to evaluate the 3-factor structure of the PEmbS-LLA compared to a potential 1-factor or 2-factor solution. For the 1-factor model, the χ^2 statistic turned out significant (χ^2_{32} = 57.76, p = .003), which was not the case for the 2-factor (χ^2_{31} = 44.44, p = .056) and 3-factor model (χ^2_{30} = 42.33, p = .067), indicating sufficient fit for the latter two models, both of which clearly showed better fit compared to the 1-factor model (χ^2_1 = 13.32, p < .001 for the 2-factor model; χ^2_2 = 15.43, p < .001 for the 3-factor model). Although there was a descriptive trend for better fit of the 3-factor model, the difference to the 2-factor model was not significant (χ^2_1 = 2.11, p = .15). Both, the 2-factor model and the 3-factor model had almost identical and sufficient values for all included fit indices (norm fit index: .95; incremental fit index: .99; Tucker–Lewis index: .98; comparative fit index: .99)[10-12], and also showed an acceptable and non-significant RMSEA value of .06 each (both $p \geq$ .31)[13]. Thus, based on the CFA, there is evidence against a single prosthesis embodiment model, but this analysis alone does not allow for preferability of either the 2-factor or the 3-factor model (both models are given in Figure S2). However, together with the exploratory factor analysis, complemented by parallel analysis, the analysis regarding the hierarchical factor structure, and not least theoretical plausibility, the 3-factor solution was favoured in the present work.

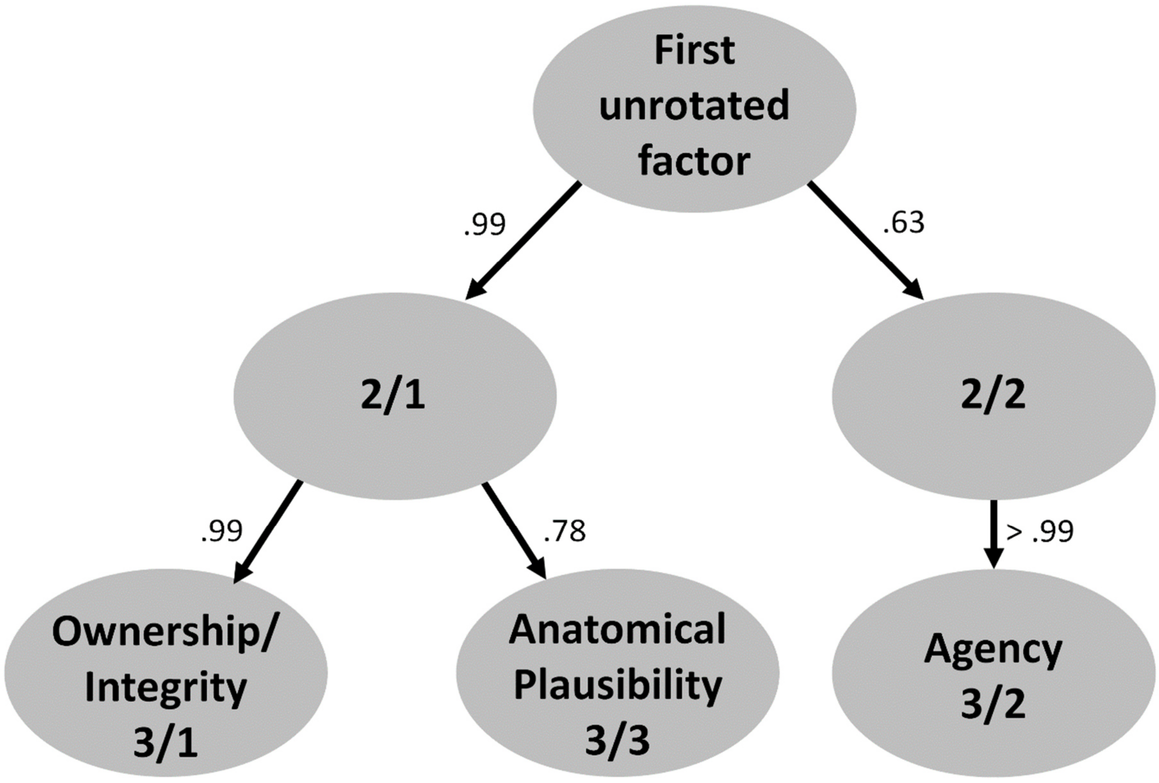

Figure S1: Hierarchical factor structure (according to Goldberg[1]) of the Prosthesis Embodiment Scale for Lower Limb Amputees. Path coefficients reflect the correlation (Pearson's *r*) of factor scores (based on the regression method).

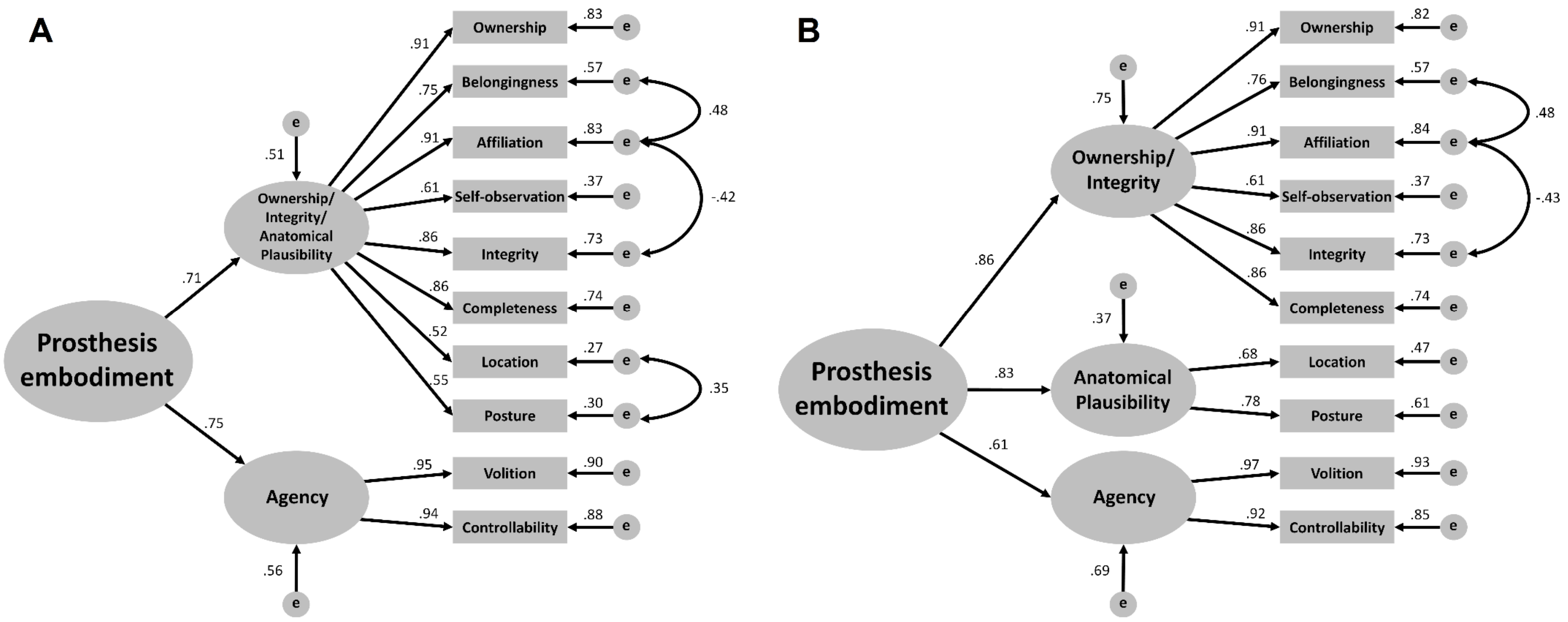

Figure S2: Confirmatory factor analysis for the 2-factor (A) and 3-factor solution (B) of the Prosthesis Embodiment Scale for Lower Limb Amputees data. Given are the standardized estimates.

Scoring of the the PEmbS-LLA sub-scales

The items identified as representing prosthesis embodiment factors were averaged (see Table S1 for the descriptive analysis). Each sub-scale's raw score was characterized by substantial negative skewness and kurtosis, and thus, the distributions significantly differed from normality according to the Shapiro-Wilk test ($W_{118} \leq .91$, all $p < .001$). Reverse square root transformation normalized the distribution only for *Ownership/Integrity* ($W_{118} = .98$, $p = .08$).

Supplementary Table S1: Characteristics of raw and transformed Prosthesis Embodiment Scale subscale scores.

	Prosthesis Embodiment Scale sub-scales					
	Ownership/ Integrity		Agency		Anatomical Plausibility	
	Raw[a]	Transf[b]	Raw[a]	Transf[b]	Raw[a]	Transf[b]
Mean	1.10	1.21	2.24	1.86	1.67	1.46
SD	1.55	0.60	1.11	0.64	1.21	0.59
Median	1.50	1.22	2.50	1.74	2.00	1.45
IQR	1.75	0.71	1.00	1.00	1.50	0.71
Skewness	-1.02	0.04	-2.20	-0.65	-1.32	0.14
SE Skewness	0.22	0.22	0.22	0.22	0.22	0.22
Kurtosis	0.37	-0.33	5.72	-0.54	1.73	-0.32
SE Kurtosis	0.44	0.44	0.44	0.44	0.44	0.44

[a] non-transformed data, potential range from -3 to +3; [b] reversed square root transformed data, back reversed, potential range from 0 to √6 (≈ 2.45); *SD* = standard deviation; *IQR* = interquartile range; *SE* = standard error

Reliability: internal consistency and test-retest stability of the Prosthesis Embodiment Scale's sub-scales

Cronbach's alpha for the *Ownership/Integrity* sub-scale was .92. Standardized Cronbach's alpha (Spearman-Brown coefficients) was .94 for *Agency* and .70 for *Anatomical Plausibility*. These values suggest excellent internal consistency for the *Ownership/Integrity* and *Agency* sub-scales, and an acceptable value for the *Anatomical Plausibility* sub-scale.[5,14]

Temporal stability was good for the *Ownership/Integrity* subscale score (r_{30} = .81 p < .001). For *Agency* (ρ_{30} = .63) and *Anatomical Plausibility* (ρ_{30} = .59, both p < .001), it was lower and not acceptable. For *Ownership/Integrity*, contextual stability was significant but not acceptable (r_{14} = .58, p = .02). For *Agency* and *Anatomical Plausibility*, the relationship between both assessments was non-significant (ρ_{14} = .49, p = .06 and ρ_{14} = .18, p = .50, respectively).

Further validation

Correlations between PEmbS-LLA subscales and both TAPES-R and LCI measures are given in Table S2.

Supplementary Table S2: Correlation between Prosthesis Embodiment Scale sub scales and clinically relevant measures.

		Prosthesis Embodiment Scale sub scales (transformed data)		
	Mdn (IQR)	Ownership/ Integrity	Agency	Anatomical Plausibility
Basic locomotor capability	4.00 (0.00)	.179	.277	.210
Advanced locomotor capability	4.00 (0.29)	.203	.323**	.227
Aesthetic prosthesis satisfaction	2.00 (0.67)	.296*	.286*	.295*
Functional prosthesis satisfaction	2.40 (0.60)	.324**	.460***	.351**
General adjustment	3.80 (0.60)	.393***	.430***	.342**
Social adjustment	3.60 (1.00)	.415***	.177	.215
Adjustment to limitation	2.80 (1.20)	.419***	.344**	.479***

Spearman correlation coefficient (ρ); n = valid number; * $p < .05$; ** $p < .01$; *** $p < .001$ (all p values Bonferroni-corrected, two-tailed)

Supplementary materials

The Prosthesis Embodiment Scale for Lower Limb Amputees (PEmbS-LLA) – German and English versions and scoring sheet

Prothesen-Embodiment-Skala für Beinamputierte (PEmbS-LLA)

□ Ich habe meine Prothese angelegt.

Anleitung: Bitte sorgen Sie dafür, dass Sie Ihre angelegte Prothese gut sehen können (z.B. durch das Tragen einer kurzen Hose). Geben Sie nun für jede der folgenden Aussagen an, wie sehr Sie dieser zustimmen oder diese ablehnen. Wenn Sie einer Aussage zustimmen, kreuzen Sie bitte eine positive Zahl an (1, 2, 3): je positiver die Zahl, desto mehr stimmen Sie der Aussage zu. Wenn Sie eine Aussage ablehnen, kreuzen Sie bitte eine negative Zahl an (-1, -2, -3): je negativer die Zahl, desto mehr lehnen Sie die Aussage ab. Die Null (0) sollen Sie nur auswählen, wenn Sie der Aussage weder zustimmen noch sie ablehnen. Antworten Sie spontan, ohne lange zu überlegen, und lassen Sie keine Aussage aus. Es gibt keine richtigen oder falschen Antworten.

Bitte betrachten Sie nun Ihre Prothese für etwa 60 Sekunden.

□ Ich habe meine Prothese etwa 60 Sekunden lang betrachtet und bin bereit fortzufahren.

		**starke Ablehnung**						**starke Zustimmung**
1.	Ich habe das Gefühl, direkt auf mein eigenes Bein zu schauen, und nicht auf eine Prothese.	-3	-2	-1	0	1	2	3
2.	Die Prothese gehört zu mir.	-3	-2	-1	0	1	2	3
3.	Ich habe das Gefühl, zwei Beine zu besitzen.	-3	-2	-1	0	1	2	3
4.	Die Prothese ist mein Bein.	-3	-2	-1	0	1	2	3
5.	Die Prothese ist ein Teil meines Körpers.	-3	-2	-1	0	1	2	3
6.	Die Stellung/Haltung der Prothese entspricht der eines echten Beins.	-3	-2	-1	0	1	2	3
7.	Ich fühle mich körperlich komplett.	-3	-2	-1	0	1	2	3
8.	Die Prothese befindet sich an der Position, an der sich mein Bein befinden würde, wenn es nicht amputiert wäre.	-3	-2	-1	0	1	2	3

Bitte stehen Sie auf und gehen Sie für etwa 30 Sekunden durch den Raum.

□ Wenn Sie nicht durchs Zimmer gehen können, kreuzen Sie hier und lassen Sie die nächsten Aussagen unbeantwortet.

□ Ich bin für etwa 30 Sekunden durchs Zimmer gegangen und bin bereit fortzufahren.

		**starke Ablehnung**						**starke Zustimmung**
9.	Die Prothese bewegt sich so, wie ich das will.	-3	-2	-1	0	1	2	3
10.	Ich habe die Kontrolle über die Prothese.	-3	-2	-1	0	1	2	3

Prosthesis Embodiment Scale for Lower Limb Amputees (PEmbS-LLA)

□ I have put on my prosthesis.

Instruction: Please make sure that you can look directly at your prosthesis (for instance, by wearing shorts). For each of the following statements, please indicate how much you agree or disagree with it. If you agree with the statement, mark one of the positive numbers (1, 2, 3): the more positive the number, the more you agree with the statement. If you disagree with the statement, mark one of the negative numbers (-1, -2, -3): the more negative the number, the more you disagree with the statement. You should select the zero (0) only if you neither agree nor disagree with the statement. Please reply spontaneously, without thinking twice, and do not skip any statement. There are no right or wrong answers.

Please look at your prosthesis for about 60 seconds.

□ I have looked at my prosthesis for about 60 seconds and I am ready to continue.

		**strongly disagree**						**strongly agree**
1.	I feel as if I was looking directly at my own leg, rather than at a prosthesis.	-3	-2	-1	0	1	2	3
2.	The prosthesis belongs to me.	-3	-2	-1	0	1	2	3
3.	It feels as if I had two legs.	-3	-2	-1	0	1	2	3
4.	The prosthesis is my leg.	-3	-2	-1	0	1	2	3
5.	The prosthesis is a part of my body.	-3	-2	-1	0	1	2	3
6.	The posture of the prosthesis corresponds to that of a real leg.	-3	-2	-1	0	1	2	3
7.	My body feels complete.	-3	-2	-1	0	1	2	3
8.	The prosthesis is in the location where I would expect my leg to be, if it was not amputated.	-3	-2	-1	0	1	2	3

Please stand up and walk around the room for about 30 seconds.

□ If you are not able to walk around the room, mark here and skip the following items.

□ I have walked around the room for about 30 seconds and I am ready to continue.

		**strongly disagree**						**strongly agree**
9.	The prosthesis is moving the way I want it to move.	-3	-2	-1	0	1	2	3
10.	I am in control of the prosthesis.	-3	-2	-1	0	1	2	3

Scoring of the Prosthesis Embodiment Scale for Lower Limb Amputees (PEmbS-LLA)

**PEmbS-LLA total score (recommended)**	**Mean of items #1-10**	___________

<u>Sub-scales, 3-factor solution</u>

Ownership/Integrity	Mean of items #1-5,7	___________
Agency	Mean of items #9,10	___________
Anatomical Plausibility	Mean of items #6,8	___________

<u>Sub-scales, 2-factor solution</u>

Ownership/Integrity + Anatomical Plausibility	Mean of items #1-8	___________
Agency	Mean of items #9,10	___________

Supplementary References

1. Goldberg, L. R. Doing it all bass-ackwards: The development of hierarchical factor structures from the top down. J. Res. Pers. 40, 347–358 (2006).
2. Byrne, B. M. Structural equation modeling with AMOS: basic concepts, applications, and programming (multivariate applications series). New York: Taylor & Francis Group (2010).
3. Bollen, K. A. Structural equations with latent variables. New York: John Wiley & Sons (1989).
4. Schermelleh-Engel, K., Moosbrugger, H., & Müller, H. Evaluating the fit of structural equation models: Tests of significance and descriptive goodness-of-fit measures. *MPR Online* **8**, 23–74 (2003).
5. Eisinga, R., Grotenhuis, M. te & Pelzer, B. The reliability of a two-item scale: Pearson, Cronbach, or Spearman-Brown? *Int. J. Public Health* **58**, 637–642 (2013).
6. Rousson, V., Gasser, T. & Seifert, B. Assessing intrarater, interrater and test-retest reliability of continuous measurements. *Stat. Med.* **21**, 3431–3446 (2002).
7. Gallagher, P., Franchignoni, F., Giordano, A. & MacLachlan, M. Trinity amputation and prosthesis experience scales: a psychometric assessment using classical test theory and Rasch analysis. *Am. J. Phys. Med. Rehabil.* **89**, 487–496 (2010).
8. Grisé, M. C., Gauthier-Gagnon, C. & Martineau, G. G. Prosthetic profile of people with lower extremity amputation: conception and design of a follow-up questionnaire. *Arch. Phys. Med. Rehabil.* **74**, 862–870 (1993).
9. Franchignoni, F., Orlandini, D., Ferriero, G. & Moscato, T. A. Reliability, validity, and responsiveness of the locomotor capabilities index in adults with lower-limb amputation undergoing prosthetic training. *Arch. Phys. Med. Rehabil.* **85**, 743–748 (2004).
10. Bentler, P. M., & Bonett, D. G. (1980). Significance tests and goodness of fit in the analysis of covariance structures. *Psychol. Bull.* **88**, 588–606.
11. Bollen, K. A. A new incremental fit index for general structural equation models. *Sociol. Methods Res.* **17**, 303–316 (1989).

12. Bentler, P. M. Comparative fit indexes in structural models. *Psychol. Bull.* **107**, 238–246 (1990).

13. Browne, M. W., & Cudeck, R. Alternative ways of assessing model fit. In: L. Bollen & J. S. Scott (eds), *Testing structural equation models*, pp 136-162, Newbury Park: Sage (1993).

14. George, D. & Mallery, P. *IBM SPSS Statistics 19 Step by Step: A Simple Guide and Reference* (Pearson, 2012).
